# Combining First-Person Video and Gaze-Tracking in Medical Simulation: A Technical Feasibility Study

**DOI:** 10.1155/2014/975752

**Published:** 2014-02-19

**Authors:** Adam Szulewski, Daniel Howes

**Affiliations:** ^1^Department of Emergency Medicine, Kingston General Hospital, Queen's University, 76 Stuart Street, Kingston, ON, Canada K7L 2V7; ^2^Critical Care Program, Department of Emergency Medicine, Kingston General Hospital, Queen's University, 76 Stuart Street, Kingston, ON, Canada K7L 2V7

## Abstract

Crisis decision-making is an important responsibility of the resuscitation team leader but a difficult process to study. The purpose of this pilot study was to explore the potential of gaze-tracking technology to study decision-making and leadership behaviours in simulated medical emergencies. We studied five physicians with a broad range of experience in a simulated medical emergency using gaze-tracking glasses. Subjects were interviewed immediately after the scenario while viewing a first-person recording of their performance with a superimposed gaze indicator. The recordings were then studied independently by two reviewers, and rated for quality and their observations collated. Portable gaze-tracking devices were found to be useful and effective tools for studying information gathering and decision-making behaviours in simulated medical emergencies. The data obtained in this study provided information about the discrepancy between what each participant looked at compared to what each participant consciously noted. Analysis of the data also identified a number of recurrent gaze patterns performed by team leaders that could be used as end-points in future research. Gaze-tracking in resuscitation medicine is a new and promising field of study. The potential to study crisis decision-making behaviours, and cognitive load, as well as differences between novice and expert team leaders is substantial.

## 1. Introduction

In recent years, there has been an increased appreciation for the importance of crisis resource management and team dynamics in the success of a medical resuscitation [1]. This change is reflected in international standardized resuscitation guidelines [2] and reflects a greater appreciation for the impact of human factors. Effective leadership is an important factor in team success [3].

Crisis decision making is an important responsibility of the team leader, but a difficult process to study. Decision making is largely an internal process that occurs rapidly at a conscious, preconscious, and automatic level; it is not visible to the outside observer and may be unknown to the decision-maker. The urgency of the crisis makes real-time interviews impossible, while post-event recollection may be inaccurate and unclear.

Eye-tracking (or gaze-tracking) technology has been used in nonmedical fields [4], particularly in the study of consumer purchasing decisions [5] and cognitive psychology [6]. Early eye-tracking devices required that the subject's head remain in a fixed position and used precise, delicate equipment. Advances in technology now permit mobile eye tracking and realistic, unencumbered interaction with the environment. Similar technology has been used to study observational behaviour in medical fields like pathology [7], radiology [8], and anaesthesia [9].

The purpose of this pilot study was to explore the potential of eye-tracking technology to study decision-making and leadership behaviours in simulated medical emergencies. We evaluated the quality of the data and explored the strengths and limitations of the technology in a dynamic environment. Finally, we sought to identify recurrent patterns that could serve as gaze behaviour end-points for future studies.

## 2. Methods

A convenience sample of five participants was chosen to represent a range of experience in resuscitation medicine. The sample included a medical student, three emergency medicine residents, and an experienced attending emergency medicine physician.

**Figure 1:**
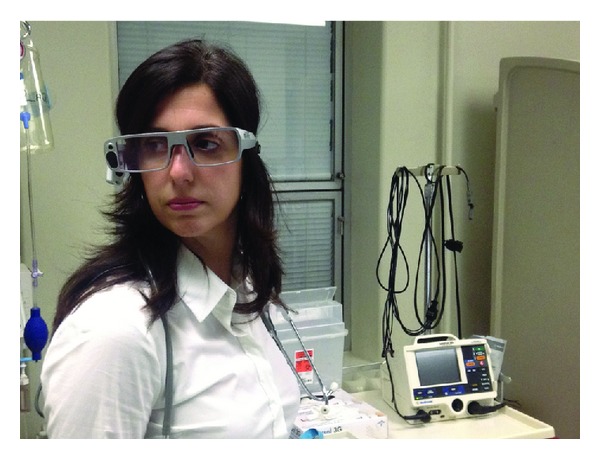
Study participant wearing portable gaze-tracking glasses.

After consenting to participation, subjects were fitted with the gaze-tracking device (Tobii Glasses Eye Tracker, Tobii Technology) (Figure 1). The device, which is worn like a pair of eyeglasses, was calibrated for each participant to nine gaze points using the manufacturer's recommended method, a process taking approximately 1-2 minutes. Next, subjects were asked to lead a medical team consisting of trained nurse and respiratory therapist actors to care for a high-fidelity simulated patient (Susie, Gaumard) in status asthmaticus for approximately 15 minutes.

**Figure 2:**
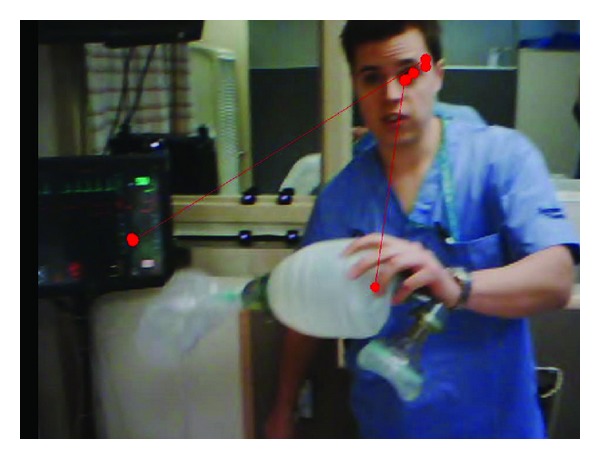
Screen shot of the first-person video recording with the superimposed dynamic gaze indicator (orange circles with connecting lines).

During the standardized simulation, the gaze-tracking device recorded first-person audio/video and gathered information about pupil position and glint location at a rate of 30 Hz. Using this data, a dynamic, real-time gaze position indicator was superimposed on the first-person video by computer software (Figure 2). The software also identified patterns of pupil movement, location, and duration of points of fixation. In addition, audio/video from three ceiling-mounted cameras was recorded on a digital system (ETC).

After completing the scenario, subjects were debriefed. While watching the recording of their performance with the superimposed gaze indicator, subjects were prompted to report internal dialogue and decision-making considerations. The audio of this interview was recorded on a separate, synchronous track with the original recording of the scenario, to allow for simultaneous playback.

The original first-person recordings were reviewed independently by two reviewers (Daniel Howes and Adam Szulewski). They rated the quality, clarity, and utility of the audio/video and gaze indicators of each session on a 10-point, anchored Likert scale. Later, each reviewer independently analyzed the final video with the subjects' debriefing narrative to identify advantages and limitations that were realized with the addition of the first-person video. The two reviewers' analyses were then collated.

Ethical approval for this study was obtained from the Queen's University Faculty of Health Sciences Ethics Review Board (approval reference number EMED-162-11; date of approval 06/12/2011).

## 3. Results

### 3.1. Findings

We found that the gaze-tracking device (with glasses and belt pack) did not limit behaviour and allowed for data collection while the subject moved around the room. Fitting and calibrating the device for each participant was easy and efficient.

The quality, clarity, and utility of the audio/video recordings were reported by both reviewers as very good (8/10) or better. The device was able to consistently discriminate participants' gaze as they looked between relatively small areas of interest. For example, it was clear from the recordings if a subject was looking at the pulse oximetry tracing versus the blood pressure reading while observing the monitor. There was some periodic loss of the gaze indicator when subjects looked to the peripheral extremes of their gaze fields as well as during blinking.

It was found that the combination of first-person video and gaze tracking (FP+G) provided information about what the participant *looked at*. The postscenario interview provided insight into what was *consciously noticed*. It was clear from the interviews that subjects may have been looking at a particular area or object without consciously “seeing” it. If a subject's working memory was occupied (trying to remember a drug dose or follow an algorithm), he/she could be blind at a conscious level to what his/her eyes were looking at.

From their standpoint, participants consistently commented on the added value of watching their own performance from a first-person perspective during the debriefing. Participants stated that this allowed them to critically analyze and evaluate their actions as a result of the added prompting that the recording provided.

The reviewers identified a number of outcome measures for future eye-tracking studies. These are outlined in Table 1.

### 3.2. Identified Limitations of the Gaze-Tracking Technology

All participants reported forgetting that they were wearing the eye-tracker moments after the scenario began and feeling that they performed naturally; however, at the completion of the scenario some reported discomfort. In longer resuscitations, this discomfort could become distracting and a reminder about the presence of the device.

Unfortunately, the eye-tracker was unable to pick up information from the peripheral field of view of participants if they looked outside of the lenses. Moreover, a slight lag in gaze data was noted when participants moved their heads quickly during the exercise. These issues have the potential to confound results.

Anatomic variation in eye shape, as well as long eyelashes, drooping eye lids, dark iris color, and corrective lenses (both glasses and contacts), has been reported to interfere with data capture [10], which could potentially result in rejection of some subjects' data. To prevent selection bias in future studies, a priori quantitative description of data quality for exclusion should be defined. This may impact sample size calculations and recruitment.

## 4. Discussion

The gaze-tracking device used in this study collected meaningful and reliable data in the dynamic environment of simulated resuscitation. In addition to the FP+G data, the software created heat plot graphs, which showed the proportion of time that participants spent looking at an object or area in their environment by colour intensity overlaid on a two-dimensional photograph of their surroundings. The particular order of points of fixation could be analyzed and then compared amongst participants to identify patterns.

The FP+G view provided useful information about the first step in decision-making—information gathering. However, it was clear from the interviews and from observing subjects' behaviour that “looking at” and “seeing” on a conscious level are very different.

Previous studies have examined the usefulness of eye-trackers in the determination of where people look and what they remember seeing. Johansen and Hansen investigated this in website design. They found that website users remembered only 70% of the elements they had looked at on a website [11]. The proportion of these “looked-at” elements that is actually remembered in a medical resuscitation is unknown, but with the help of eye-trackers, we should be able to identify this fraction.

The FP+G recordings provided insight into team dynamics and interpersonal interactions within the team. By providing the opportunity to see the room from the perspective of the team leader, the recordings allowed the observer to appreciate a more honest “feel for the room” than what the team leader or participants might normally be willing to share in traditional debriefings.

Finally, the recordings were effective in helping subjects recall their internal cognitive processing during the resuscitation. Indeed, without this playback technology, much of this internal processing might be lost, and the potential to learn from it would disappear as quickly as subjects' memories of the scenario faded.

## 5. Conclusions

Portable gaze-tracking devices are useful tools for studying decision making in medical emergencies in a novel fashion. In this experiment, we identified a number of parameters that could be used as end-points in future research and were able to introduce gaze tracking as a means to better understand crisis decision making in medicine.

The potential for further research with gaze tracking in resuscitation medicine is substantial. Comparisons between novice and expert team leaders could lead to a better understanding of how leadership skills develop. A more detailed understanding of the behaviours of clinicians with more expertise might have potential as a quantitative evaluation tool in medical education. In addition, an examination of subtle eye movements may serve as an indicator of emotional stress. Moreover, changes in pupil size (which can be measured with the gaze-tracker) have been shown to be associated with cognitive load [12], which could potentially be used as a quantitative marker for increased ability and experience.

Although the study of resuscitation using gaze-tracking technology in the simulated medical environment is promising, further study in real-life scenarios is critical to developing a more complete understanding of medical crisis decision making.

## Figures and Tables

**Table 1:** Potential outcome measures for future studies that were identified during data review.

Outcome variable	Potential data to be gathered	Notes
Prioritization of information gathering	(i) Where do subjects look first upon entering a medical crisis situation? (ii) Gaze tracking for the first 30 seconds of a medical crisis.	(i) If used in a simulation lab, the subjects will need to be equally familiar with the lab environment (ii) What subjects look at may not be the same as what they see

Devalued information	Where did the subject not look?	(i) Not looked at must be not seen (i.e., an area that the subject did not look at cannot have been consciously noted) (ii) Strong indicator of what is not valued

Dwell time analysis	(i) Software-generated heat plots (ii) Specific target times (e.g., total time with gaze focused on a specific area)	(i) Good indicators of where gaze is targeted over the majority of the time during the resuscitation (ii) Comparison of key targets between groups may identify behaviour trends

Specific gaze behaviours	(i) Scanning	(i) Checking the environment, patient indicators, or team members. Suggests that there is free working memory for this task.
	(ii) Confirmation	(ii) Seeking eye contact from team members. May also be done verbally.
	(iii) Checking-in	(iii) Looking to ensure that an order has been carried out.
	(iv) Perching	(iv) Gaze tends to focus or “perch” on corners and edges of objects in participants' field of view when they are actively thinking or recalling information.

Cognitive load and stress indicators	(i) Pupil Dilation (ii) Frequency of microeye movements	Influenced by both cognitive load and emotional load: may be difficult to separate influence.
